# Dosimetry methods and clinical applications in peptide receptor radionuclide therapy for neuroendocrine tumours: a literature review

**DOI:** 10.1186/s13550-018-0443-z

**Published:** 2018-08-29

**Authors:** Daphne Merel Valerie Huizing, Berlinda Jantina de Wit-van der Veen, Marcel Verheij, Marcellus Petrus Maria Stokkel

**Affiliations:** 1 Department of Nuclear Medicine, Netherlands Cancer Institute, Plesmanlaan 121, 1066 CX Amsterdam, The Netherlands; 2 Department of Radiation Oncology, Netherlands Cancer Institute, Plesmanlaan 121, 1066 CX Amsterdam, The Netherlands

**Keywords:** Dosimetry, Systemic radiation therapy, PRRT, Absorbed dose, Neuroendocrine tumours

## Abstract

**Background:**

The main challenge for systemic radiation therapy using radiopharmaceuticals (SRT) is to optimise the dose delivered to the tumour, while minimising normal tissue irradiation. Dosimetry could help to increase therapy response and decrease toxicity after SRT by individual treatment planning. Peptide receptor radionuclide therapy (PRRT) is an accepted SRT treatment option for irresectable and metastatic neuroendocrine tumours (NET). However, dosimetry in PRRT is not routinely performed, mainly due to the lack of evidence in literature and clinical implementation difficulties. The goal of this review is to provide insight in dosimetry methods and requirements and to present an overview of clinical aspects of dosimetry in PRRT for NET.

**Methods:**

A PubMed query including the search criteria dosimetry, radiation dose, peptide receptor radionuclide therapy, and radionuclide therapy was performed. Articles were selected based on title and abstract, and description of dosimetric approach.

**Results:**

A total of 288 original articles were included. The most important dosimetry methods, their main advantages and limitations, and implications in the clinical setting are discussed. An overview of dosimetry in clinical studies regarding PRRT treatment for NET is provided.

**Conclusion:**

Clinical dosimetry in PRRT is feasible and can result in improved treatment outcomes. Current clinical dosimetry studies focus on safety and apply non-voxel-based dosimetry methods. Personalised treatment using sophisticated dosimetry methods to assess tumour and normal tissue uptake in clinical trials is the next step towards routine dosimetry in PRRT for NET.

**Electronic supplementary material:**

The online version of this article (10.1186/s13550-018-0443-z) contains supplementary material, which is available to authorized users.

## Background

Ionising radiation is already effectively used to treat cancer for over a century. In this respect, several sources of radiation with different features and clinical applications are available. External beam radiation therapy (EBRT) delivers high-energy ionising radiation from outside the body, whereas brachytherapy involves sealed sources internally placed in proximity to the target [1, 2]. This manuscript focuses on the third type of therapeutic radiation: systemic radiation therapy (SRT), also known as radionuclide therapy. Like the localised EBRT and brachytherapy, SRT results in a palliative or curative effect by ingestion or systemic administration of a molecular complex containing a β^−^- or α-emitting isotope [2, 3]. Although SRT has been used for decades, it has gone through a revival with the introduction of targeted radiolabelled antibodies and small molecules. Examples are somatostatin analogues directed towards the somatostatin receptor and ligands to target the prostate-specific membrane antigen (PSMA) to treat neuroendocrine tumours (NET) and prostate cancer, respectively. This type of SRT is often referred to as peptide receptor radionuclide therapy (PRRT) for NET and peptide radionuclide ligand therapy (PRLT) for prostate cancer [4, 5]. The widespread introduction of PRRT for NET in the USA and in Europe was stimulated by the completion of the phase III NETTER-1 study. In this study, safety and effectiveness of Lutetium-177 (^177^Lu) DOTATATE was evaluated in metastatic midgut NET patients and resulted in market registration [6]. A meta-analysis by Kim et al. shows that the average disease control rate after treatment with PRRT is 82%. However, response rates are lower: 18–44% based on RECIST criteria and 7–37% based on SWOG criteria [7].

The key to any type of radiation therapy is to ensure sufficient absorbed dose into tumour lesions, while minimising the burden to healthy tissues. Treatment planning and dose verification using dedicated software to optimise the balance between tumour control probability (TCP) and normal tissue complication probability (NTCP) is considered standard of care in the field of radiation therapy [8]. Still, when applying SRT, most centres employ a ‘one-size-fits-all’ approach for the amount of radioactivity administered similar to chemotherapeutic regimes, rather than calculating individualised internal dose estimates [9–11]. Dosimetry in SRT may refer to either the estimation of radioactivity that needs to be administered to achieve a desired absorbed dose (i.e. planning or pre-treatment dosimetry) or estimation of the absorbed radiation dose after administration of the radiopharmaceutical (i.e. verification or post-treatment dosimetry) [12]. The absorbed dose can be estimated if information on patient-based radiopharmaceutical kinetics, biodistribution, isotope characteristics, anatomical geometry and tissue densities are present [2, 12]. In this respect, TCP and NTCP values used in EBRT cannot be directly applied to SRT, as the absorbed dose in both therapies does not result in the same cell killing effect. EBRT delivers high dose rates in a controlled setting using an external irradiation source, whereas in SRT, radioactive sources delivers a low and continuously decreasing dose rate for longer time [13, 14]. In a series of recent published editorials, experts in the field of nuclear medicine, physics and dosimetry provided their vision on the usability of individual dosimetry for SRT [11, 15, 16]. Proponents state that the amount of administered radioactivity should be ‘as high as reasonably possible’ to achieve an optimal treatment outcome. This requires, however, personalised analysis as interpatient pharmacokinetic variations are large. Furthermore, they suggest that dosimetry-based optimisation should be added to the registration, in addition to fixed treatment schemes, to allow for clinical dosimetry [11, 15]. Opponents state that dosimetry has a role in radiopharmaceutical development and safety, but its clinical use is not evidence-based. They emphasise caution when transferring from the well-established and safe empirical dosage schemes towards the complex, time-consuming, and non-standardised dosimetry approaches [16]. Regardless of this ongoing discussion, the 2013/59/Euratom statement of the European Union stipulates that radiotherapeutic procedures should be individually planned and verified [17].

This literature review discusses the main dosimetry methodologies for PRRT in NET, their drawbacks and appropriate use followed by a structured overview of clinical applications. Additionally, imaging quantification, kinetic modelling and the biologically effective dose are briefly touched upon.

## Review

### Search strategy

The search strategy was designed to identify published peer reviewed articles that cover dosimetry in a clinical or technical research setting concerning PRRT for NET. Studies published between July 2006 and July 2017 were included. A PubMed search was performed using the following terms: “PRRT”[All Fields] OR “nuclear therapy”[All Fields] OR “radionuclide therapy”[All Fields]) AND (“dosimetry”[All Fields] OR “radiation dose”[All Fields]). Additional filter included the English language and letters, commentaries, editorials, case reports, reviews and preclinical studies were excluded.

### Selection for full-text review

Articles identified based on the search strategy were subdivided into two groups based on title and abstract: (1) technical description of dosimetry or (2) clinical dosimetry in PRRT for NET. Technical articles should at least describe the imaging methodology, data type (digital simulation, phantom or patient data), isotope and dosimetry methodology. Clinical articles should focus on PRRT and had to describe the radiopharmaceutical, administered radioactivity, patient population, dosimetry methodology, imaging approach and absorbed dose estimates.

### Results

**Fig. 1:**
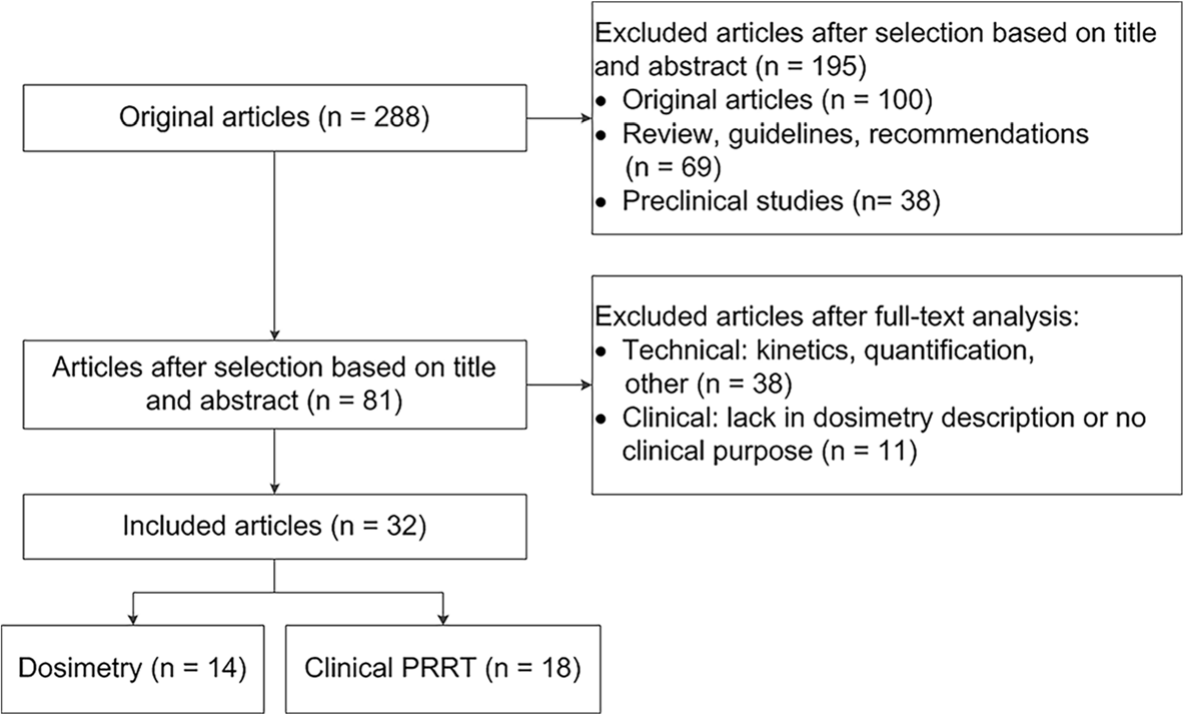
Selection workflow of the search query

In total, 288 unique articles were identified from the structured search, including 181 original articles, 38 preclinical articles and 69 reviews/guidelines/recommendations. Initial selection based on title and abstract excluded 195 articles; after analysis of full-text articles, only 32 out of 288 articles fulfilled the selection criteria (14 technical, 18 clinical). The detailed selection workflow is shown in Fig. 1, and a summary of the included articles is provided in the Additional file 1: Tables S1 and S2. In the following four consecutive sections, Monte Carlo simulation, the Medical Internal Radiation Dose (MIRD) formalism and *S* values, dose kernels and local energy deposition are discussed. An overview of all methods is provided in Table 1.

**Table 1:** Overview of dosimetry methods

Method	Assumptions	Advances	Drawbacks	Clinical application
Monte Carlo simulation	Simulation of certain number of particles. Manual particle energy cut-off values	Very accurate, includes tissue density heterogeneities and cross-fire dose	Many simulation parameters. Long-calculation times	Not applicable for clinical routine. Calculation of *S* values and dose kernels
*S* values	Homogeneous radioactivity distribution in tissue	Fast, easy, commonly used and generally accepted	Based on reference phantoms, mean absorbed dose per tumour or organ	Organs and lesions without superimposition. Toxicity studies
Dose kernels	Homogeneous radioactivity distribution within one voxel, infinite homogeneous tissue density	DVH and isodose lines, patient-specific	Calculated for each radionuclide, not tissue specific. Mean absorbed dose per voxel	Patient-specific voxel-based tumour and normal tissue dosimetry
Local energy deposition	All energy is absorbed in the source voxel	Fast	Not suitable for photons	Primarily for β^−^- and α-emitters

#### Method 1: Monte Carlo simulations of radiation transport

Monte Carlo (MC) simulation is based on an iterative statistical process to estimate random pathways and interactions of particles in three dimensions, allowing for voxel-level absorbed dose estimations [18]. Numerous input parameters are required for an accurate simulation, including scattering and absorption behaviour, medium characteristics and the number of simulated primary particles. In general, MC simulations are quite extensive taking tissue penetration depth, energy loss, bremsstrahlung photons and cross-fire dose into account [19, 20]. The cross-fire dose refers to irradiation of a structure by its surroundings and is especially relevant for isotopes with γ-emission due to the longer path length through tissue compared to β^−^- and α-particles or auger electrons. Voxel-based methods that incorporate cross-fire dose will result in improved dose estimations [19]. Different MC simulator toolkits are nowadays available (see Additional file 1).

The main advantages of MC simulations are the capability to account for an inhomogeneous radioactivity distribution, induction of secondary particles (often γ-radiation), transitions between tissue types, and patient-specific organ and lesion geometries [21, 22]. Modern quantitative imaging techniques (PET/CT and SPECT/CT) are as input for MC simulations and provide information on anatomical geometry, tissue densities, heterogeneities and (non-uniform) distribution patterns. To date, full MC simulations are not recommended for routine clinical use due to complex calculations and relative long computational times (roughly 3 h for ~ 10 million simulations) [23–25]. In most articles, MC simulations in PRRT are used to validate new faster algorithms for specific assumptions on activity distributions, absorption, cross-fire, and tissue transitions [19, 20, 22, 24, 25].

#### Method 2: MIRD formalism and S values

The MIRD formalism, as developed by the Medical Internal Radiation Dose (MIRD) committee of the Society of Nuclear Medicine (SNM), was originally designed to estimate average radiation doses to patients as received by radiopharmaceuticals [26]. The system provides a framework to assess mean absorbed doses to organs, tissues, voxels and cellular compartments [27]. The formalism presumes deposition of energy from source volume *s* in target volume *t* described by (*t* ← *s*) [28–30]. Quantitative imaging at multiple time points are required to create the time-activity curve, from which the cumulative radioactivity ($\overset{\sim }{A}$) in a volume of interest is calculated.

The MIRD formalism can be adopted using *S* values (mGy MBq^− 1^ s^− 1^), which describe the mean absorbed dose in the target volume per unit cumulative radioactivity in the source. *S* values have been determined for various isotopes using MC simulations [29, 31, 32]. The source-to-target distance, tissue density, target mass and the radionuclide emission spectrum impact the *S* value. Nowadays, *S* values are available for specific tissues and radiopharmaceuticals in software packages [33].

Homogeneous distribution of radioactivity within organs and standardised organ mass are assumed when using *S* values as described in MIRD pamphlet no. 5 (1975) and no. 11 (1969) [29, 31]. Traditionally, simple mathematical humanoid models, including standardised organs with fixed dimensions and spheres of different volumes to represent tumours, were used for dosimetry analysis while assuming infinite homogeneous media with soft tissue density [31]. The latest MIRD/ICRP (International Commission on Radiological Protection) voxel-based anthropomorphic phantoms are specified for male, female and children of different ages [28]. Although patient-specific organ masses can be derived from diagnostic imaging, adjustments for position, tissue inhomogeneity and shape of organs are not yet feasible [22, 34].

*S* value dosimetry is accessible for clinical use due to relative simple, quick algorithms that only requisite sequential 2D imaging to estimate activity distributions and the use of average organ characteristics [30]. This technique has become the standard dosimetry method for pharmaceutical studies, despite the previous mentioned assumptions [35–38]. Tumour dosimetry is possible, although cross-fire dose is not taken into consideration and tumour lesions are assumed to be spherical [39]. In recent literature, *S* values are applied in treatment safety monitoring [13, 35, 40, 41]. Furthermore, dosimetric analysis using *S* values is often used as a reference for new dosimetry methodologies [42–44].

#### Method 3: dose kernels for voxel dosimetry

Quantitative 3D imaging techniques like PET/CT and SPECT/CT visualise non-uniformities within organs and tumours on a voxel-level. MIRD pamphlet no. 17 (1999) provides voxel-based dosimetry in analogy with the MIRD formalism using voxel *S* values (VSV). VSV are specified for specific isotopes and voxel dimensions, calculated using MC simulations [22, 45]. Each voxel is considered an individual uniform source and neighbouring voxels as uniform targets [24, 46]. Mean absorbed dose calculations per voxel are performed using a dose kernel matrix (mGy MBq^− 1^ s^− 1^), resulting in a voxel-by-voxel dose map [47]. Dose estimates may differ depending on the MC code. However, variances are often within a few percent and are not considered relevant in a clinical setting [21, 34, 44].

**Fig. 2:**
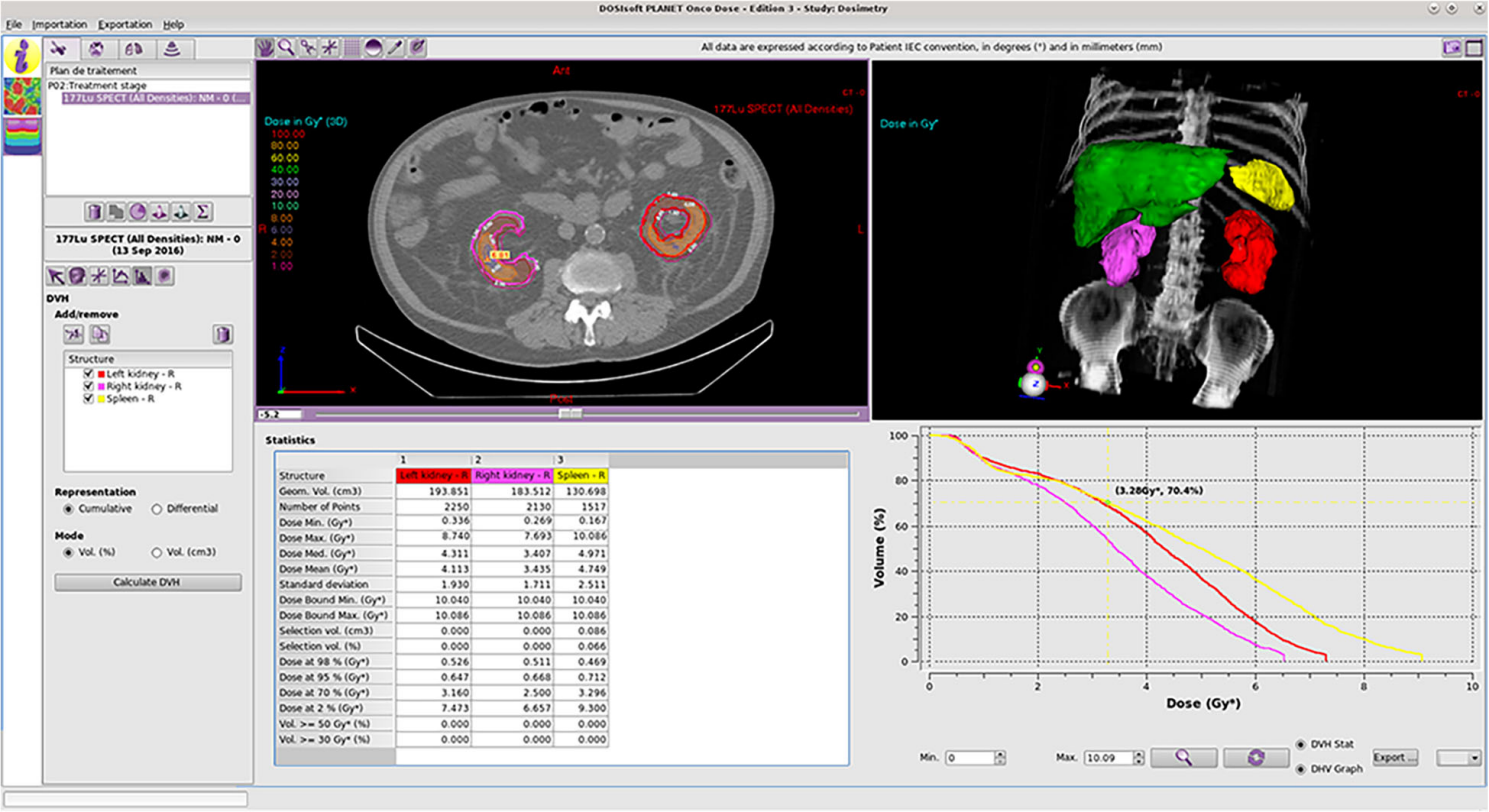
Example of kidney dosimetry after PRRT in PLANET® Dose. Isodose lines superimposed on anatomical images provide a detailed view (upper left), whereas the summary table (lower left) and dose-volume histogram (lower right) enable a quick assessment. Courtesy of DOSIsoft SA

Advantages of dose kernel dosimetry are the ability of handling inhomogeneous radioactivity distributions at organ or tumour level [24]. Furthermore, 3D dose distributions enable visualisation of isodose lines and dose-volume histograms (DVHs) for radiobiological assessment, as shown in Fig. 2 [44, 46]. This approach is quickly gaining popularity in centres that have sufficient SPECT/CT or PET/CT capacity and that want to perform patient-centred dosimetry, as the calculation time is about 10 s per case [24]. Still, it has to be stated that full MC simulations should be used when different tissue densities (other than soft tissue) or inter-voxel heterogeneities are deemed relevant [19, 22, 34].

In literature, dose kernel research focuses on density corrections, methods to speed up the calculation and comparison of different kernels [24, 48, 49]. In addition, in-house software tools with VSV are widely developed [19, 25, 43, 50].

#### Method 4: local energy deposition

In addition to the three main pillars of dosimetry in nuclear medicine therapy, local energy deposition method for dosimetry calculations is applied. Here, all energy is assumed absorbed in the voxel of origin. This theory holds true for certain α- and β-particles or auger electrons, but does not apply for γ-emissions or secondary photons due to the longer penetration depth. However, if one is primarily interested in assessing certain parts of the radionuclide emission spectrum, then this method is fairly accurate for a quick analysis like in toxicity studies [19, 51, 52]. Other methods should be considered for radionuclides with high γ-yield, and therefore, a high contribution of cross-fire dose [19, 20]. This γ-irradiation cross-fire effect between tumour and organ or between organs is considered marginal in PRRT [53, 54]. Yet, cross-fire of β^−^-particles due to internalisation of the labelled peptides between cells is significant [55].

### Clinical dosimetry in PRRT for NET

Already in 2011, the EANM Dosimetry Committee published a ‘good practice’ document on dosimetry reporting, stimulating structured reporting of scientific results with specific attention for instrumentation and protocols [56]. Details concerning (gamma) camera type, including collimator, number of heads and crystal thickness should be noted. Furthermore, acquisition settings, camera calibration procedures, and image processing and analysis should be described in detail when performing dosimetry. The pharmacokinetic section should include the number of time points, type of time-activity curve fitting and interpolation. Finally, the source of *S* values, tumour dosimetry methodology and origin of organ mass need to addressed. Surprisingly, most clinical dosimetry articles as discussed in this review did not provide all details on image acquisition and kinetic modelling.

**Fig. 3:**
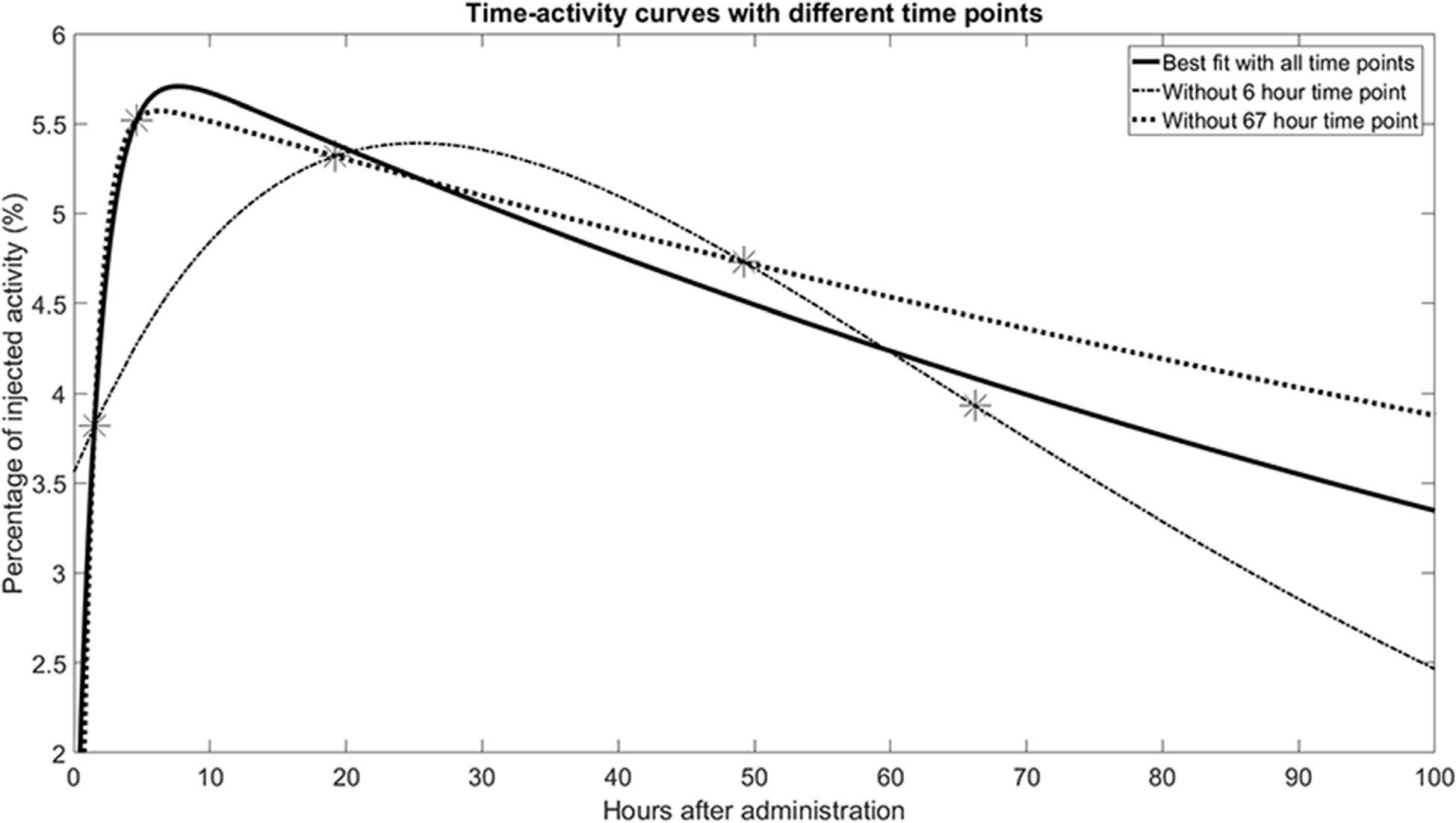
Example of time-activity curve fitting. Optimal curve fitting using all five time points is represented by the solid black line. The dash-dot line demonstrates what happens if an early time point is not performed; the maximum activity is underestimated. An overestimation of activity in the tail of the curve could occur when a late time point is omitted (dotted line). Adapted from [37]

Out of the 18 selected clinical articles on PRRT in NET, 11 articles used planar gamma imaging, 4 articles used SPECT/CT and 3 articles combined both techniques (see Additional file 1). Sandström et al. recommended the use of SPECT/CT for tumour dosimetry, since this modality enables improved quantification accuracy compared to planar gamma imaging [57]. Variations concerning the number of time points for kinetic modelling were observed, as three up to seven time points were described. The importance of sequential imaging, and especially inclusion of late time points (> 48 h post-injection for small molecules as used in PRRT), is indicated by multiple studies. The addition of late time points may affect the cumulative radioactivity with ~ 5% [52, 53, 58–60]. Figure 3 visualises the effect on time-activity curve fitting while omitting an early or late time point. The MIRD formalism with *S* values from the OLINDA/EXM software package or tabulated dose factors (DF) acquired from the RADAR website were applied in all but two articles. One article performed the local energy deposition method, while the other one applied VSV.

Most of the included clinical studies were designed for safety monitoring; 12 articles focussed on the kidney and 4 articles on bone marrow (BM) toxicity. Regarding kidney toxicity, multiple publications recommended individualised kidney dosimetry due to the high interpatient variability of absorbed doses [13, 52, 54, 61]. Dose-response relations were presented in various papers, for example, Schuchardt et al. described the association between mean absorbed doses and kidney toxicity [41]. An individualised treatment schedule for PRRT with standardised kidney absorbed doses of 23 Gy was proposed by Del Prete et al., which resulted in increased tumour doses while limiting renal toxicity in simulated personalised treatment schemes. Hence, a personalised PRRT schema based on tumour dosimetry would have led to higher mean absorbed doses to the kidney. This method is currently evaluated in a prospective clinical trial [40]. Most studies adopted kidney absorbed dose thresholds between 23 and 27 Gy [9, 36, 40, 62]. Bergsma et al. suggested to increase the kidney maximum absorbed dose up to 28 Gy [36]. This is supported by the fact that half of the patients do not reach 23 Gy after 4 cycles of 7.4 GBq ^177^Lu-DOTATATE [57]. Individual dosimetry would have enabled additional cycles of PRRT in these patients. Moreover, dosimetry was used to evaluate the kidney dose delivered by different ^177^Lu-labelled peptides [63].

In conjunction to kidney dosimetry, individualised dosimetry to assess BM toxicity is indicated and a dose limit of 2 Gy is accepted [58, 64]. BM dosimetry can be performed using both imaging and non-imaging approaches [65]. Sequential blood samples are often used to estimate the self-dose to the BM using blood kinetics [10, 58, 64, 65]. In most patients, self-dose is the most dominant source of BM irradiation [9]. However, estimation of the cross-fire effect from large organs (mainly the kidney, liver and spleen) and bone metastases require quantitative imaging [58]. Whole-body scintigraphy is essential in this respect, as the field-of-view of SPECT/CT is limited, and the activity in the remaining body cannot be estimated [54]. Alternatively, urine samples can be used to estimate the activity in the remainder of the body [58]. Yet, collecting urine and blood samples is labour intensive for both the patient and hospital employees. Imaging is often performed using three to four time points, where blood sampling five up to eight samples was described [9, 58, 64]. Clinical BM dosimetry studies were based on imaging, urine and blood sampling data. In addition, a novel method using only planar imaging to estimate the BM dose without blood sampling is available [62].

Tumour dosimetry was described in nine clinical studies, and an association between absorbed tumour dose and therapy outcome was observed in two studies [53, 59]. Simulated personalised PRRT based on the absorbed dose by the kidney resulted in a 1.47-fold higher tumour dose, what could lead to increased therapy response in a clinical situation [40]. Furthermore, the relation between uptake on diagnostic imaging and dosimetry was studied [66].

## Conclusions

This review provides a structured overview of modern dosimetry methods in PRRT and their current clinical applications, potentials and limitations in NET treatment. In the last decade, many steps have been made towards personalised PRRT in NET using dosimetry. The incentive to perform dosimetry to optimise PRRT for individual patients is of importance, as limited data about maximum tolerable dose to normal tissue and optimal tumour dose is still known [67]. For instance, three phase II studies did not reach the maximum tolerated administered activity, while reporting response rates between 7 and 54% [68–70]. Though we are far from achieving high response rates, most patients treated with PRRT are assumed palliative patients, so optimising treatment implicates extending a patient’s life in relative good health. The individual optimal number of cycles and administered activity can be determined using tumour and normal tissue dosimetry. On the other hand, population data can be used to determine for example the average maximum absorbed dose to the kidney and the influence of fractionated treatment [71]. Nevertheless, several hurdles need to be overcome prior to routine clinical implementation.

### Dosimetry protocols

The included clinical articles implemented various dosimetry protocols. Most studies applied S value based dosimetry from difference sources, despite recommendations to use voxel-based approaches [57, 72]. In our opinion, dose kernels are the most appropriate method for dosimetry in PRRT. The main reason is that heterogeneous organ and tumour uptake can be taken into consideration, yet the method is more practicable compared to the complicated MC simulations [24, 46]. Furthermore, the number of time points for post-therapy imaging was diverse. Current guidelines do not propose specific time points, but address the essence of dispersed post-therapy imaging in case of slow radiopharmaceutical washout [73]. Two to three time points in both the uptake and excretion phase are recommended [30]. Nevertheless, for wide clinical implementation four up to six time points are unsuitable for clinical departments as it is time consuming. Recent research has focussed on optimising the number of time points, for example by only using one late time point [74]. Maaβ et al. applied pharmacokinetic models based on individual and population information to estimate kidney and tumour uptake with different sampling schedules [75]. For the kidneys, the use of only the 4 h and 2 days time point allowed for sufficient time-integrated activities estimates. This approach was not appropriate for tumours, as the uptake variability between patients is large, so for tumour dosimetry, one has to stick to at least two early and two late time points. Finally, the importance of late time point imaging to estimate the tail of the curve was pointed out by multiple clinical papers [52, 53, 58–60].

In addition, it is essential to provide a complete overview of the applied methodology, as is pointed out by the EANM [56], in order to compare and share knowledge. For dosimetry opponents, the lack of well-designed studies to demonstrate the value of individual dose planning and verification is the main reason not to deviate from empirical posology schemes [16]. Nonetheless, the joint IAEA, EANM and SNMMI practical guideline on PRRT for NET states that patient-specific dosimetry can provide valuable information and dosimetry could contribute to PRRT optimization [38]. Therefore, it is essential that radiopharmaceutical companies and regulatory agencies allow for dosimetry-based individual treatment schedules and not only fixed administrations [15].

### Safety considerations

In research, most clinical studies focus on therapy safety while using fixed activities and intervals between cycles. This results in a lack of clinical evidence for patient-based dosimetry. A number of studies observed patient-specific dose-effect relationships concerning tumour lesions and kidney or bone marrow dose, which could result in increased response and decreased toxicity rates [35, 59, 61, 76]. Dosimetry can be used to assess individual risks for renal toxicity, when combined with 3D imaging and patient-specific volumes and masses [72, 76]. In most clinical evaluations, the maximum kidney dose is fixed to 23 Gy, which is the 5% probability of nephrotoxicity 5 years after irradiation as used in EBRT [53, 77]. However, this threshold might not be appropriate for PRRT [36]. The recent prospective study of Garske-Román et al. shows a response rate of 30.9% based on RECIST criteria in patients who have received 23 Gy to the kidney [78]. In this group, only one patient showed grade 4 nephrotoxicity 3 years after PRRT and no grade 3 toxicity was observed. This fact supports the hypothesis that currently, most patients are undertreated if the number of cycles and amount of administered radioactivity is based on the 23 Gy absorbed dose by the kidney. The biologically effective dose (BED) can be of interest, as it indicates the absorbed dose with the same biological effect independent from the irradiation source. Adjustments for BED calculations for PRRT are suggested, due to the low dose rates and inhomogeneous irradiation during PRRT compared to EBRT [72]. Differences in BED and treatment schedules are explored for PRRT using both ^90^Yttrium (^90^Y) and ^177^Lu [79]. The BED can be determined in vitro using the linear-quadratic model, which describes cell survival after direct DNA damage. Indirect damage due to the bystander effect could occur due to the long irradiation times and relatively low dose rates in PRRT. Irradiated cells may induce radiation effects in surrounding cells by cell-to-cell contact and the abscopal effect. This bystander effect implies the release of mediators to induce oxidative stress in neighbouring cells [80]. Further research on radiobiology and clinical dosimetry studies, preferably by randomised clinical trials, should be combined to optimise PRRT [15, 16].

### Technical imaging considerations

Clinical dosimetry is challenging due to the balance between clinical and technical requirements. Sequential post-therapy 3D imaging and subsequent image processing to provide voxel-based dosimetry is time-consuming and is, for now, reserved to a limited number of specialised centres. Whereas planar gamma images suffer from superimposition, what complicates accurate determination of radioactivity concentrations. The addition of at least one SPECT acquisition can contribute to quantification optimisation, while providing a time-efficient imaging protocol [39, 81]. When sequential imaging is limited to planar gamma imaging in clinical routine, the conjugate-view method with one additional SPECT/CT (hybrid approach) will increase accuracy of delineation and quantification [81]. Still, both planar and SPECT imaging suffer from the γ-imaging drawbacks such as limited spatial resolution due to scattered photons, collimator septal penetration by high-energy photons, attenuation, and statistical noise in low count rates [30, 82]. A comparison between quantitative imaging based on only planar imaging, the hybrid approach and multi SPECT/CT imaging showed a significant difference between all three methods [83]. Multi whole-body planar and hybrid dosimetry resulted in an overestimation of the mean absorbed kidney dose compared to multi SPECT/CT of 1.6 and 1.2 times, respectively. From a quantitative perspective, it is recommended to perform at least one SPECT/CT acquisition to improve quantification accuracy, provided that the calibration factor is determined according to guidelines [82]. Techniques like CT-based attenuation correction are strongly advised in SPECT/CT and PET/CT to improve quantification. Likewise, scatter correction and iterative reconstruction techniques may further improve image quality and quantitative assessment [84]. A harmonisation initiative as is provided by the EANM (EANM Research Ltd., EARL) could aid in improvements of multicentre quantitative gamma imaging [85]. A Dutch quantitative SPECT initiative already performed a multicentre analysis for ^99m^Technetium studies [86].

Image processing using relatively small volumes of interest (VOI) of ~ 4 ml could decrease the time in preparation for dosimetry in solid organs. Manual whole-organ segmentation is time-intensive, and kidney volume determined by thresholding is unstable and often changes over time. Studies have shown that this small VOI method results in less than 5–10% difference in absorbed dose compared to segmentation based on anatomical information or thresholding [40, 54, 57]. As regards to tumour dosimetry, we suggest to segment the full lesion instead of small VOI segmentation. Tumours show more often heterogeneous uptake compared to healthy tissues. Small VOI segmentation might therefore over- or underestimates the total lesion dose.

### Dosimetry software considerations

Many dosimetry methods as described in the technical articles use in-house developed algorithms, limiting the translation of results to other centres. Within the EU, software tools are considered medical devices when they are used for clinical decision-making, through which FDA/CE-approval is a prerequisite for implementation. At the time of writing, only a handful of FDA/CE-approved systems are commercially available. OLINDA/EXM® v1.0 developed by the RADAR-group was one of the first registered tools and has recently been commercialised by Hermes Medical Solutions (OLINDA/EXM® v2.0, Stockholm, Sweden) [33, 87]. Other just recently CE-marked commercial systems are PLANET® Dose (DOSIsoft, Chachan, France) and Simplicit90Y™ (Mirada Medical Ltd., Oxford, UK). All tools initially focused on either 2D or 3D dose planning or verification, but are gradually providing 2D/3D dosimetry solutions for PRRT to enable a hybrid dosimetry approach. Up to now, no studies are published comparing absorbed dose outcomes of these systems.

### Proposal for a clinical dosimetry workflow in PRRT

**Fig. 4:**
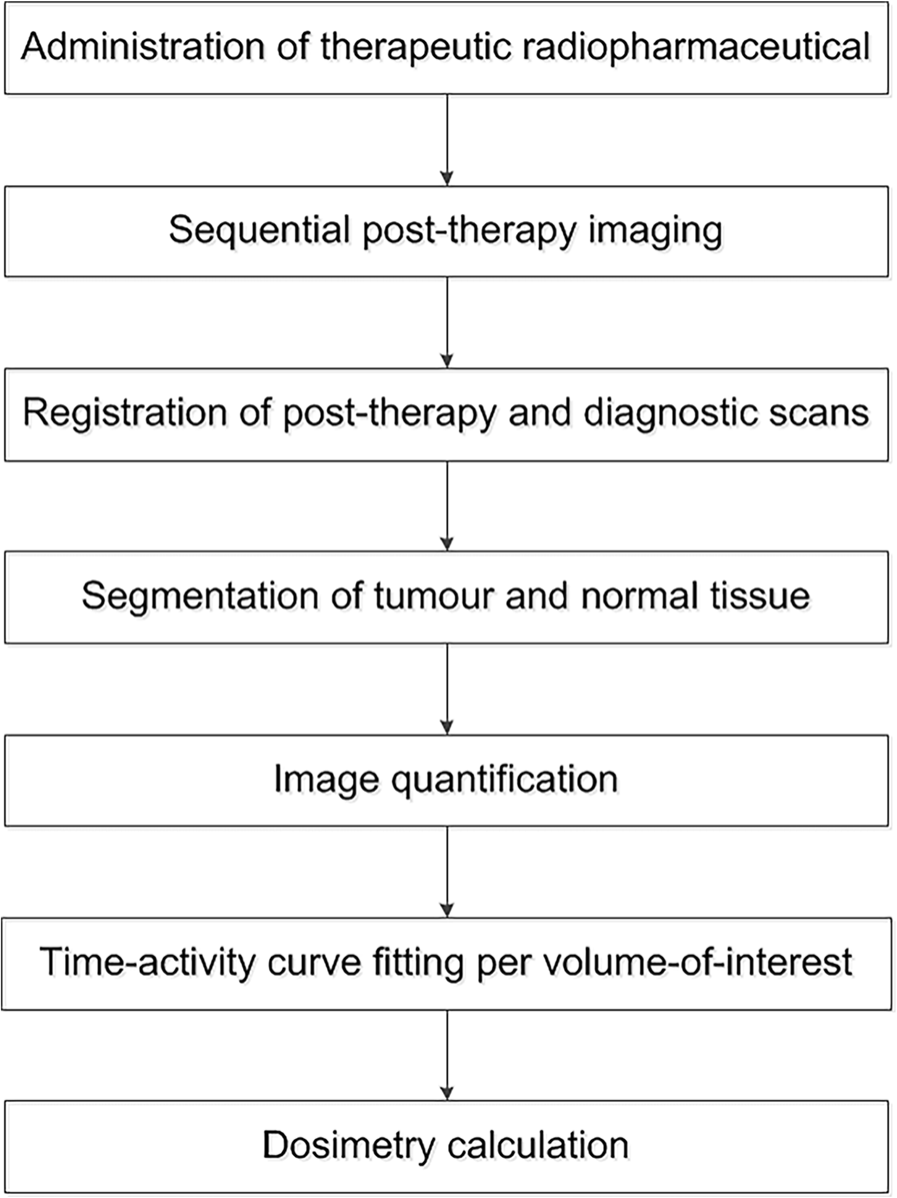
Example of a schematic workflow for clinical dosimetry in PRRT

Any dosimetry workflow for PRRT in NET depends on a few critical steps, see Fig. 4. Sequential imaging is essential to create a proper time-activity curve and determine the cumulative activity in a volume of interest [75, 88]. We recommend to use three late time points, with the latest time point at least later than two effective half-lives of the radiopharmaceutical, to accurately fit the tail of the time-activity curve [30].

In our opinion, the hybrid 2D/3D approach provides sufficient quantification accuracy while patients do not have to go through sequential long SPECT/CT acquisitions. Multiple authors share this point of view [81, 83]. Still, one has to take in mind that quantitative SPECT errors between 5 and 18% are noted in phantom experiments [82, 89].

In our current clinical experience, adjustments of the administered activity in PRRT are based on haematological assessment. In case of decreased blood parameters, the administered activity will be reduced from the standard 7.4 GBq to either 3.7 or 5.5 GBq. Based on the aforementioned uncertainties in dose estimations and practical reasons; we feel that adjustments to the administered activity based on dosimetry should be adapted in steps of ~ 1 GBq.

Optimisation of tumour control and normal tissue toxicity is of main concern in individual dosimetry for PRRT, thus the dosimetry methodology should meet that demand. MC simulations should be avoided in standard clinical setting due to the inherent complexity. *S* values are highly accessible since 3D imaging is not a requisite; however, the method is not designed for inhomogeneous activity distributions [31]. Dose kernel approaches are nowadays available to handle heterogeneous radioactivity distributions at voxel-level for individual tumour or normal tissue dose planning or verification. An advantage of voxel-based methods is the ability to calculate DVHs and show isodose lines, which can assist in treatment optimisation [24]. Post-therapy visualisation of the actual delivered tumour dose allows for clinical correlation with the local tumour response, even in a multicentre setting. This approach is expected to contribute to PRRT prescription of administered activity, as tumour-type based response and expected toxicities can be tailored.

Finally, large multicentre trials are essential to take big steps in data collection, improvement of quantitative imaging across all centres performing PRRT and harmonisation of dosimetry methodologies. The need for randomised controlled clinical trials is acknowledged by both physicians and physicists [11, 14]. A certain trial requires well-organised harmonised training to perform quantitative imaging, time-activity curve fitting and dosimetry calculations from a technical perspective. Proper trials could further aid in optimisation from a radiobiological point of view, as current literature contains a large variety of dosimetry methodologies [90]. If a large consortium for dosimetry in PRRT can be established, the future will be bright for NET-patients.

#### Availability of data materials

Data sharing not applicable to this article as no datasets were generated or analysed during the current study.

## Additional file

Additional file 1: **Table S1.** Technical articles concerning dosimetry approaches. Table S2 Clinical studies using dosimetry in PRRT in NET. (DOCX 30 kb)

## Abbreviations

^177^Lu: ^177^Lutetium
^90^Y: ^90^Yttrium
BED: Biologically effective dose
BM: Bone marrow
Bq: Becquerel
CT: Computed tomography
DVH: Dose-volume histogram
EANM: European Association of Nuclear Medicine
EBRT: External beam radiotherapy
Gy: Grey
IAEA: International Atomic Energy Agency
ICRP: International Commission on Radiological Protection
MC: Monte Carlo
MIRD: Medical Internal Radiation Dose
NET: Neuroendocrine tumour
NTCP: Normal tissue complication probability
PET: Positron emitted tomography
PRRT: Peptide receptor radionuclide therapy
SNM: Society of Nuclear Medicine
SPECT: Single-photon emission computed tomography
SRT: Systemic radiation therapy
TCP: Tumour control probability
VSV: Voxel *S* values
