# Supplementary material for: Dosimetry methods and clinical applications in peptide receptor radionuclide therapy for neuroendocrine tumours: a literature review
Source: EJNMMI Res. 2018 Aug 29;8:89. doi: 10.1186/s13550-018-0443-z (PMC6115319; doi:10.1186/s13550-018-0443-z)
Supplement: Supplementary file 1 — Table S1. Technical articles concerning dosimetry approaches. Table S2 Clinical studies using dosimetry in PRRT in NET. (DOCX 30 kb) [file 13550_2018_443_MOESM1_ESM.docx]

Supplement: Overview of included articles

Table 1. Technical articles concerning dosimetry approaches.

| Article | Isotope | Imaging | Methodology | Software | Assumptions | Limitations |
| --- | --- | --- | --- | --- | --- | --- |
| Beranato 2016[49] | ^111^In, ^177^Lu, ^90^Y | SPECT/CT and phantom | VSV | STRATOS | VSV require validation before clinical use to ensure correct dose calculations. | The use of different VSV can lead to different absorbed doses. All radionuclide emissions should be taken into account in the VSV calculation. |
| Dieudonné 2012[24] | ^177^Lu, ^131^I, ^90^Y | SPECT/CT | MC simulation, VSV | 3D-RD, VoxelDose | Density corrections for VSV result in better dose estimation and increased similarity to the dose calculated by MC simulation. | Density corrections for VSV have the most influence on voxel-level, less for organ and tumour dosimetry. |
| Fernandez 2013[48] | ^177^Lu, ^90^Y, ^131^I | Not applicable | High resolution VSV | MCNPX | Based on interpolation, fits and resampling, VSV calculated for small voxel sizes can be adapted for larger voxel sizes. | Calculation times for the initial high resolution VSV are long. |
| Grassi 2013[42] | ^177^Lu | SPECT/CT | VSV, S values | VoxelMed, STRATOS,  OLINDA/EXM | Effective half-life is het most accurate method to estimate the tail of the time-activity curve. | Registration and curve fitting play essential roles in the performance of dosimetry software. |
| Hippelaïnen 2015[19] | ^177^Lu | SPECT/CT | Semi-MC simulation | In-house developed | Local absorption of electrons and simulation of photons. | Underestimation of absorbed dose can occur in healthy tissue when omitting cross-fire dose. |
| Jackson 2013[43] | ^177^Lu | SPECT/CT | VSV, S values | VRAK, OLINDA/EXM | VSVs account for geometrical aspects in case of cross-fire, while S values do not. | S values might be more suitable for hollow organs and VSV for solid structures. |
| Kletting 2015[50] | ^177^Lu  ^90^Y  ^131^I | PET/CT,  SPECT/CT,  WB scintigraphy | VSV, S values | NUKDOS, OLINDA/EXM, RADAR | VSV are taken from literature or interpolation. | VSV are only nuclide specific, not tissue density specific. Larger deviation with high energy particles (^90^Y) and in lung tissue. |
| Kost 2015[18] | ^177^Lu, ^131^I, ^90^Y, ^111^In | SPECT/CT, PET/CT | MC simulation | VIDA | Tumour lesions were assumed as spheres of tissue-equivalent material in medium of scatter and water. | Requires sequential SPECT/CT acquisitions. |
| Lanconelli 2011[21] | ^89^Sr, ^90^Y, ^131^I, ^153^Sm, ^177^Lu, ^186^Re, ^188^Re | Not applicable | VSV | DOSXYZnrc | VSV for bone and soft tissue separately using monochromatic sources with 25 million simulated particles. | VSV work only when the assumption of homogeneous tissue is reliable, otherwise MC simulations should be performed. |
| Lin 2012[23] | ^131^I | Not applicable | Voxel-by-voxel activity sample algorithm | EGSnrc | Each voxel with activity is a homogenous, independent voxel with all equal size. Contribution of each voxel is weighted. | Differences between other MC methods because of different modelling parameters. |
| Loudos 2009[45] | ^153^Sm | PET/CT, SPECT/CT | VSV | GATE | VSV calculation uncertainty decreases for bone tissue but increases for small voxel sizes. | Dose kernels are limited to the simulated voxel sizes and distances from the source voxel. |
| Mirzaei 2013[33] | ^177^Lu | WB scintigraphy | S values using the MIRD formalism | Hybrid Dosimetry | Kinetic modelling and dosimetry analysis is combined in one software package. | Not yet 3D dosimetry to introduce patient-specific organ masses; is currently time-consuming to determine organ border. |
| Saeedzadeh 2012[44] | ^131^I | Zubal phantom | MC simulation, S values | GATE, MIRDOSE3, DPM | GATE searches for neighbouring voxels of the source voxel within the same medium. | S values do not provide a DVH, from which tumour responses could be predicted. |
| Sanchez-Garcia[25] | ^99m^Tc as surrogate for ^90^Y | SPECT/CT | Collapsed cone, compared with MC simulation and dose voxel kernels | In-house developed, MCNP6, VoxelDose | Mean effective tissue density can correct for heterogeneous tissue density. Homogenous activity distribution in voxel. | Soft tissue, cortical bone or lung tissue are the options for tissue density corrections. |

SC = scatter correction, AC = attenuation correction, BED = biological effective dose, EUD = equivalent uniform dose, VIDA = Voxel-Based Internal Dosimetry Application, VRAK = Voxelized Registration and Kinetics, NURBS = Non-Uniform Rational B-Spline digital human phantom, GATE = Geant4 Application for Tomographic Emission. Different MC simulation codes: Geant4 (Conseil Européen pour la Recherche Nucléaire (CERN), Geneva, Switserland), Monte Carlo N-particle (MCNP) code (Los Alamos National Laboratory, Los Alamos, New Mexico, USA), and Electron Gamma Shower (EGSnrc) code (National Research Council Canada, Ottawa, Ontario, Canada).

Table 2. Clinical studies using dosimetry in PRRT in NET.

| Article | Radiopharmacon | Number of patients | Post-therapy imaging | Time points | Dosimetry method and software | Tissue of interest | Main conclusions |
| --- | --- | --- | --- | --- | --- | --- | --- |
| Bergsma 2016 [36] | ^177^Lu- and ^90^Y-DOTATATE | 407 | WB planar | 1, 3, and 7 days | S values from RADAR | Kidney | The maximum dose to the kidney as derived from EBRT for ^90^Y-DOTATATE therapy does not seem to account for ^177^Lu-DOTATATE. |
| Bergsma 2016 [64] | ^177^Lu-DOTATATE | 32 | WB planar & blood sampling | 24, 96, and 168h | S values from OLINDA/EXM | Bone marrow | A dose limit of higher than 2 Gy to the bone marrow should be appropriate for PPRT. |
| Van Binnebeek 2014 [35] | ^111^In-pentretreotide  as surrogate for ^90^Y-DOTATOC | 50 | WB planar | 15 min, 4, 24, 48h | S values from OLINDA/EXM | Kidney | BED of 37 Gy is a good threshold for PRRT, to prevent renal toxicity in combination with renal protection. |
| Del Prete 2017 [40] | ^177^Lu-octreotate | 36 | SPECT/CT | 4, 24 and 72h | S values from OLINDA/EXM | Kidney and tumour lesions | Personalized PRRT is possible and leads to increase tumour doses and limited kidney toxicity. |
| Ezziddin 2012 [66] | ^177^Lu-DOTATATE and -DOTATOC | 21 | WB planar | 1, 2, 4 days | S values from OLINDA/EXM | Target tumour lesions without superimposition | SUV measurements on PET/CT could predict mean absorbed dose. |
| Ezziddin 2013 [59] | ^177^Lu-DOTATATE | 27 | WB planar | 1h, 3h, 1, 2, 3-4, 6-8, 10-15d | S values from OLINDA/EXM | Tumour lesions, liver, kidney, soft tissue without superimposition | Tumour-to-kidney dosimetry ratio may indicate morphologic tumour response in early cycles. |
| Forrer 2009 [58] | ^177^Lu-DOTATATE | 15 | WB planar, SPECT/CT and blood sampling | Three time points between 24 and 168h | S values from OLINDA | Bone marrow | Individual bone marrow absorbed dose calculated is needed to optimize individual amounts of radioactivity. |
| Gleisner 2015 [60] | ^177^Lu-DOTATATE | 7 | WB planar | 1, 24, 96, 168h, 5-10 weeks | S values from OLINDA | Total body, liver and tumour lesions | The contribution of data point at 5 – 10 weeks is 5-6% of the cumulative activity. |
| Hänscheid 2017 [74] | ^177^Lu-DOTATATE and -DOTATOC | 29 | WB planar | 1-4h, 1, 2 and more than 4 days | MIRD schema | Kidney, liver, spleen and tumour lesions | A single measurement four days after therapy could approximate the mean absorbed dose. |
| Hindorf 2007 [13] | ^90^Y-DOTATOC | 30 | SPECT/CT | Three time points | S values from RADAR | Whole-body, tumour lesions and kidney | High interpatient variability, low intrapatient variability. |
| Ilan 2015[53] | ^177^Lu-DOTATATE | 24 | SPECT/CT | 24, 96, 168h | S values from OLINDA/EXM | Tumours lesions | Correlation between tumour absorbed dose and tumour reduction on CT. |
| Kulkarni 2013 [63] | ^177^Lu-DOTATATE and –DOTATOC | 22 | WB planar | 0.5, 3, 20-24, 44-48, 68-72h | S values from OLINDA/EXM | Kidney | DOTATOC delivers a significant lower dose to the kidney. |
| Sandström 2010 [54] | ^177^Lu-DOTATATE | 30 | WB planar & SPECT/CT | 1, 24, 96, 168h | Dose correction factors from RADAR | Kidney | Kidney is dose-limiting organ, 28/30 patients show decrease uptake in bone marrow. Individualized dosimetry in essential. |
| Schuchardt 2013 [41] | ^177^Lu-DOTATATE,  -DOTATOC and -DOTANOC | 278 | WB planar | 0.5, 3, 20, 44, 72h | S values from OLINDA/EXM | Whole-body, normal tissue, kidney, spleen, tumour lesions | Calculation of the mean absorbed doses to tumours and critical organs could predict toxicity on individual basis. |
| Sundlöv 2017 [62] | ^177^Lu-DOTATATE | 51 | WB planar &  SPECT/CT | WB: 1, 24, 48 or 96 and 168 h.  SPECT/CT: 24h | VSV from LundADose | Kidneys | Individualized dosimetry based on the BED calculated after dosimetry is safe. |
| Svensson 2015 [52] | ^177^Lu-DOTATATE | 33 | WB planar &  SPECT/CT | WB: 1h, 1, 2, 7d  SPECT/CT: 24h | Local energy deposition | Kidney | Large variation in absorbed kidney dose between patients, late data points are necessary for better estimations, CV method is accurate. |
| Svensson 2016 [61] | ^177^Lu-DOTATATE | 46 | WB planar | 2, 24, 48, 168 h | S values from RADAR | Bone marrow | Bone marrow dosimetry can be performed using 2D planar imaging and thresholding. |
| Wehrmann 2007 [10] | ^177^Lu-DOTATATE and -DOTANOC | 69 | WB planar and blood sampling | WB planar: 3, 20, 44, and 68h | S values from OLINDA/EXM | Whole-body, normal tissue, kidney, spleen, tumour lesions, bone marrow | Large standard deviation in inter- and intrapatient variability indicates for personalized dosimetry. |
